# Assessing Lexical Psychological Properties in Second Language Production: A Dynamic Semantic Similarity Approach

**DOI:** 10.3389/fpsyg.2021.672243

**Published:** 2021-09-23

**Authors:** Kun Sun, Xiaofei Lu

**Affiliations:** ^1^Department of Linguistics, University of Tübingen, Tübingen, Germany; ^2^Department of Applied Linguistics, The Pennsylvania State University (PSU), University Park, PA, United States

**Keywords:** lexical psycholinguistic properties, word embeddings, semantic similarity, second language proficiency, dynamic approach

## Abstract

Previous studies of the lexical psycholinguistic properties (LPPs) in second language (L2) production have assessed the degree of an LPP dimension of an L2 corpus by computing the mean ratings of unique content words in the corpus for that dimension, without considering the possibility that learners at different proficiency levels may perceive the degree of that dimension of the same words differently. This study extended a dynamic semantic similarity algorithm to estimate the degree of five different LPP dimensions of several sub-corpora of the Education First-Cambridge Open Language Database representing L2 English learners at different proficiency levels. Our findings provide initial evidence for the validity of the algorithm for assessing the LPPs in L2 production and contribute useful insights into between-proficiency relationships and cross-proficiency differences in the LPPs in L2 production as well as the relationships among different LPP dimensions.

## Introduction

The lexical proficiency of second and foreign language (L2) learners^1^ plays a critical role in their overall language proficiency. While the definition of lexical proficiency continues to evolve, L2 researchers generally conceive it as encompassing vocabulary breadth and depth knowledge as well as the accessibility of core lexical items (e.g., Meara, 2005; Crossley et al., 2011b; Zhang and Lu, 2014). In the backdrop of this conceptualization, previous L2 vocabulary studies have used various types of measures to assess L2 learners’ lexical proficiency and/or the properties of their lexical usage. The first type is that of lexical diversity, often measured using the type–token ratio (TTR) or one or more of its transformations, such as the corrected TTR or the D measure (e.g., McKee et al., 2000; Kubát and Milička, 2013). Another type is that of lexical sophistication, often measured with reference to lexical frequency, with the assumption that greater use of less frequent words may indicate higher lexical proficiency (e.g., Lu, 2012). These types of measures pertain to vocabulary breadth knowledge. Studies that focus more on vocabulary depth knowledge have used measures that pertain to word meaning and/or use, such as collocations (e.g., Wolter and Gyllstad, 2011; Zhang and Lu, 2015) and lexical relationships of polysemy and hypernymy (e.g., Crossley et al., 2009, 2010).

Recent notions of lexical proficiency also increasingly attend to the psycholinguistic properties of words that affect word processing and learnability, such as word concreteness, imageability, and familiarity (Crossley et al., 2011a). While lexical psycholinguistic properties (LPPs) have been extensively explored in first language (L1) studies (Kuperman et al., 2012; Brysbaert et al., 2014), they have been the focus of relatively few L2 studies (Salsbury et al., 2011; Crossley et al., 2011a; Crossley and Skalicky, 2019). The few relevant L2 studies generally assessed the degree of an LPP dimension of an L2 corpus by computing the mean of the rating norms of unique content words in the corpus that are covered by a database of rating norms for that LPP dimension. This approach does not take word frequency into account, and the sole reliance on databases of rating norms means that it may suffer coverage problems, as many words are not represented in such databases. More critically, this static approach may not precisely reflect the actual degree of a given LPP dimension of different L2 corpora. This is because the approach assumes that the same word is used with the same degree of that LPP dimension in different corpora, while in fact L2 learners with different proficiency levels may perceive its degree differently and use it in different ways.

In L1 research, the degrees of different LPP dimensions for individual words have commonly been obtained through subjective normative ratings by a large group of L1 speakers (Bradley and Lang, 1999; Brysbaert et al., 2014). Attempts to gauge L2 learners’ ratings of the LPP dimensions of individual words, which are rare, would necessarily need to control for lexical proficiency level, as learners’ perceptions of these dimensions could change, making such attempts potentially more costly than those with L1 speakers.

In light of the gaps and limitations of extant research of LPPs in L2 learners discussed above, the present study extends a semantic similarity algorithm to estimate the degree of different LPP dimensions in L2 production. Using word embeddings drawn from a corpus, this algorithm first assesses the approximate degree of a given LPP dimension for individual words in the corpus by computing their semantic similarity to a small set of seed words selected from a database of normative ratings for that LPP dimension and then estimates the degree of the LPP dimension for the entire corpus from the information about individual words. As will hopefully become clear below, this algorithm bypasses the limitations of the static algorithm used in the previous research. We use the algorithm to analyze the degree of five different LPP dimensions of several sub-corpora of the Education First-Cambridge Open Language Database (EFCAMDAT2) representing L2 English learners at different proficiency levels. We report initial evidence for the validity of the algorithm for assessing the LPPs in L2 production and discuss our findings pertaining to between-proficiency relationships and cross-proficiency differences in the LPPs in L2 production as well as the relationships among different LPP dimensions.

## Background

### LPP Dimensions and Relevant Databases of Rating Norms

A large number of LPP dimensions have been found to affect lexical learning. In this section, we briefly review the conceptualizations of the five dimensions examined in the current study. Three of the most heavily researched dimensions are *concreteness*, *imageability*, and *familiarity*. Concreteness refers to how abstract or concrete a word is or the degree to which it can be experienced by our senses (concrete vs. abstract; e.g., the word *apple* is more concrete than *hope*), imageability represents the degree of ease in constructing a mental image of the word (imageable vs. unimageable; e.g., the word *mountain* is more imageable than *perception*), and familiarity represents how familiar a word is to the learners or how commonly it is experienced (familiar vs. unfamiliar; e.g., the word *dog* may be more familiar to learners than *resolution*; Wilson, 1988). The other two dimensions examined in the current study are *age of acquisition* (AoA) and *semantic size*. AoA indicates the age at which a word is initially acquired, often assessed by asking adults to estimate when they first learned a word in its spoken or written form (e.g., the word *water* may be acquired earlier than *prosperity*; Brysbaert, 2017). Semantic size is a measure of magnitude as expressed in either concrete or abstract terms: For concrete objects, this corresponds to their physical size (e.g., the word *ball* has a larger semantic size than *seed*); for abstract concepts, this may depend on the context or affective associations (e.g., *a big moment*, *a small problem*; Sereno et al., 2009; Yao et al., 2013).

Various databases of rating norms now exist for different LPP dimensions. Such databases were usually constructed by asking a large number of L1 speakers to rate different words for different dimensions on a scale. For example, Brysbaert et al. (2014) collected the rating norms for the concreteness of 40,000 English word lemmas by over 4,000 respondents recruited from Amazon Mechanical Turk’s crowdsourcing website, all of whom self-identified as native speakers of American English residing in the USA. Each participant was given one or more lists of words and was asked to rate each word using a five-point rating scale going from most abstract to most concrete. In terms of the number of words rated, previous studies have constructed large databases for concreteness and age of acquisition (Kuperman et al., 2012; Brysbaert et al., 2014), but the databases for other dimensions have been relatively small. This scenario changed with the recent release of the Glasgow norms (Scott et al., 2019), which contain subjective rating norms for the five LPP dimensions discussed above, with 5,500 words rated for each dimension by 100 native speakers of English from the University of Glasgow.

From a psycholinguistic perspective, the LPPs of words can affect lexical recognition, processing and learning by L1 speakers and/or L2 learners (Balota et al., 2007). For example, L1 speakers have been found to perform better on various tasks involving concrete words than those involving abstract words, including word recognition, recall, lexical decision, pronunciation, and comprehension (Gee et al., 1999; Yao et al., 2018). This pattern of learning of concrete vs. abstract words can be explained by the distributed memory representation framework, which predicts that concrete words share more conceptual features with each other than abstract words, resulting in better associative performance (Van Hell and de Groot, 1998). L1 speakers have also been found to recognize words acquired earlier in life and words referring to larger things (i.e., words with larger semantic sizes) more quickly than those acquired later and those referring to smaller things (i.e., words with smaller semantic sizes; Cortese and Khanna, 2007; Scott et al., 2019). With the incremental nature of lexical acquisitions, L1 and L2 learners are likely to acquire more frequent, common, or familiar words earlier (Schmitt and Meara, 1997; Schmitt, 1998). Some studies have shown that L2 learners acquire concrete words earlier than abstract words (Crossley et al., 2009; Salsbury et al., 2011) and that more imageable words make up better candidates for keyword techniques than less imageable words in L2 vocabulary learning (Ellis and Beaton, 1993).

As L2 learners’ vocabulary knowledge increases incrementally (both in general and in terms of their knowledge of the full range of meanings of individual words), their perceptions of the LPPs of words are likely to evolve as their proficiency and vocabulary knowledge increase. More specifically, their perceptions of the LPPs of an individual word may be adjusted as their knowledge of the meanings and uses of the word become more comprehensive and precise, and the changes in the ways in which they use the word may reflect changes in their knowledge and perceptions. For instance, as a learner’s understanding of the full range of literal and metaphorical meanings of a word more precisely, the learner’s perception of the “semantic size” of the word could be adjusted as well (e.g., the word *egg* referring to mosquito or ostrich eggs, and the word *group* referring to groups with a few or millions of members).

The argument that the perceptions of LPPs of words are not static for L2 learners at different levels of language proficiency finds support in findings of a few recent studies that attended to the interaction between lexical properties and L2 proficiency level in examining the effects of lexical properties on L2 perception and vocabulary acquisition. For example, Sawada (2019, p. 55 and 56) asked a group of Japanese learners of English to perform a lexical memorization task, in which they tried to memorize eight target words by listening to and repeating them, and a lexical retrieval task, in which they listened to the same 8 target words and 24 filler words (twice per word in random order) and subsequently judged whether they heard in the previously completed lexical memorization task or not. The results showed that although both intermediate and advanced L2 learners recognized highly familiar words faster than less familiar ones, they differed significantly in “the ability to judge among the highly familiar words in the perception and recognition process of speech.” The author concluded that “the subjective evaluation of word familiarity by L1 speakers may not be completely identical to L2 lexicon” and that proficiency level significantly affects L2 learners’ performances regarding the lexical properties of words. De Wilde et al. (2020, p. 352) investigated the effects of several word-related variables on L2 receptive vocabulary learning. They reported significant interactions between proficiency and each of the following four word-related variables: frequency, concreteness, AoA, and cognateness, indicating that L2 learners at different proficiency levels may perceive the LPPs of the same words differently. Specifically, they found that higher proficiency learners are more open to L2-related variables, while lower proficiency learners may rely more on their L1 knowledge, particularly cognate guessing, that is, “guessing the meanings of words based on similarities with known cognates.” We argue below that these studies motivate the dynamic approach to assessing the LPPs in L2 production that takes into account potentially differential perceptions across L2 proficiency levels.

### Measurement of LPP Dimensions in Previous L2 Studies

A few studies have examined the degree of different LPP dimensions in L2 corpora using two tools. Salsbury et al. (2011) used Coh-Metrix (Graesser et al., 2004) to assess changes in the degree of concreteness, familiarity, imageability, and meaningfulness of the content words in a longitudinal corpus that contained 99 oral texts produced by six English as a second language (ESL) learners over a one-year period. Here, word meaningfulness refers to “how associated a word is to other words” (Salsbury et al., 2011, p. 344), and words with high meaningfulness score are those that evoke many word associations (e.g., the word *cup* may evoke associations with *coffee*, *plate*, *saucer*, etc.). Using repeated measure ANOVAs, they found significant differences in the degree of concreteness, imageability, and meaningfulness of the content words used by the ESL learners over time, but not familiarity. Crossley and Skalicky (2019) pointed out that Salsbury et al.’s (2011) study had a relatively small sample size and did not account for individual variation. In a replication of that study, they used the Tool for the Automatic Analysis of Lexical Sophistication (TAALES; Kyle et al., 2018) to examine changes in the same psycholinguistic properties in a longitudinal corpus containing 167 transcripts of naturalistic conversation produced by 50 ESL learners over a four-month period. Their analysis yielded similar results to those reported by Salsbury et al. (2011) regarding changes in these lexical properties of learners’ productive vocabulary over time, and they thus concluded that Salsbury et al.’s findings “can be extrapolated to a different setting, subjects, and times” (Crossley and Skalicky, 2019, p. 401). These two pioneering studies contributed highly useful insights into the nature of the development of several LPP dimensions in ESL learners over time.

The algorithm used by the Coh-Metrix and TAALES searches for unique content words in a longitudinal corpus of texts produced by L2 learners, retrieves the rating norms for those words in a database, such as the MRC Psycholinguistic Database (Coltheart, 1981), which contained 150,837 words with up to 26 linguistic and psycholinguistic attributes for each word, and finally calculates the mean of the rating norms of those words covered by the database. Such an algorithm has three potential limitations. First, it averages the LPP dimension scores of content word lemmas and does not take into account of the frequencies of the lemmas in the corpus. As Hills and Adelman (2015) argued, lemmas that are more frequent in the corpus may contribute more to the overall concreteness/abstractness of the corpus and should thus be weighted higher than lower-frequency words. Second, it does not cover all words in the corpus: Content word coverage depends on the database, and function words were left out. While content word coverage may improve as larger databases become available, full coverage will likely remain unattainable. Ratings norms for various categories of function words (e.g., pronouns, determiners, prepositions, and modal verbs) are now included in recent databases for such LPP dimensions as concreteness, imageability, and AoA (Kuperman et al., 2012; Brysbaert et al., 2014; Scott et al., 2019), indicating the importance of including function words in the analysis of LPP dimensions as well. For example, in Brysbaert et al.’s (2014) database, the preposition *about* has a much lower concreteness rating than *under* (1.77 vs. 3.45). In Scott et al.’s (2019) database, the preposition *after* has a much lower imageability rating than the noun *abdomen* (2.743 vs. 6.235). Third, and more importantly, the algorithm takes a static approach to assessing the degree of LPP dimensions in different L2 corpora. As evidenced in the findings from recent studies on the interaction between L2 proficiency level and lexical properties discussed above (Sawada, 2019; De Wilde et al., 2020), the assumption that a word is perceived to have the same degree of any LLP dimension by all learners or used with the same degree of any LPP dimension across all L2 corpora may be problematic. For example, learners who have acquired metaphorical uses of the preposition *in* (e.g., *in essence*) would likely perceive the concreteness of the word differently from those who use it in its literal, concrete sense only (e.g., *in the box*). The static approach falls short of capturing such potentially different perceptions and usages.

To overcome these limitations, the current study extends a novel semantic similarity algorithm to estimate the degrees of different LPP dimensions in L2 corpora, drawing upon recent success in applying this algorithm to the investigation of diachronic change of concreteness in language. While the analysis of diachronic change differs from our analysis of cross-proficiency change in various obvious ways (e.g., the focus on native vs. L2 data), the similar focus on change over time (i.e., historical time periods vs. learning time) makes the success in using the algorithm in analyzing diachronic change relevant to our analysis. Extending the analytical scope of the two pioneering studies reviewed above, we use this new algorithm to examine the differences among L2 English learners at different proficiency levels as well as the relationships among different LPP dimensions.

### A Semantic Similarity Approach to Assessing Diachronic Change of Word Concreteness

One of the earliest algorithms for assessing diachronic change of word concreteness was proposed by Hills and Adelman (2015). Different from the algorithm used in Coh-Metrix and TAALES, this algorithm estimates the degree of concreteness of a corpus by computing a weighted average of the concreteness ratings of the words in the corpus, with weights based on the frequency of the words in the corpus. However, it suffers the same coverage problem mentioned previously for Coh-Metrix and TAALES. In addition, the approach taken by the algorithm is still static, as it relies on a fixed set of concreteness rating norms and does not account for historical fluctuations of word meanings.

Recently, Hamilton et al. (2016b) proposed the Sentiment Propagation (SentProp) algorithm for quantifying semantic change based on the notion of word embeddings, i.e., vectors representing co-occurrences of each word with other words. Specifically, this algorithm models historical fluctuations of word meanings by constructing word embeddings in each time-period and measuring how an individual word’s embedding shifts over time. Using this method, the authors detected many known semantic shifts of individual words (e.g., the word *nice* moving away from the meaning of *refined* toward that of *pleasant* around 1900). Snefjella et al. (2019) subsequently used this algorithm to assess diachronic change of word concreteness. The details of this algorithm are discussed and illustrated in the Methodology section, but we note here that, as a semi-supervised algorithm (see, e.g., Abney, 2007), it requires concreteness rating norms of a small set of seed words and estimates the degree of concreteness of each word in the corpus by computing its semantic similarity to the seed words. The semi-supervised approach is especially appropriate for the task given the availability of reliable seed words from existing databases on the one hand and the absence of fully labelled training data for all words on the other. The mechanism for computing semantic similarity from seed words constitutes a core component for semi-supervised algorithms in general. With the label propagation framework adopted for this purpose, Hamilton et al. (2016a) achieved state-of-the-art performance on inducing historical sentiment lexicons that document changes in word polarity over time (e.g., the change of *terrific* changed from a negative word to a positive word). Compared to the algorithm used in Coh-Metrix and TAALES, this algorithm not only takes word frequency into account but, because of its semi-supervised nature, effectively eliminates the coverage problem by analyzing all words in the corpus, including function words. More importantly, the approach taken by the algorithm is a *dynamic* one, in that the degree of concreteness of each word is allowed to vary across sub-corpora representing different time periods. Snefjella et al. (2019) reported significant, strong correlations (*ρ*=0.70, *p*<0.001) between concreteness estimates produced by the algorithm and human ratings of concreteness in Brysbaert et al. (2014). As their goal was to “estimate generic trends in the historical evolution of concreteness,” their analysis focused on changes in the overall concreteness scores of sets of words in different time periods. For example, for the set of word types found in all historical sub-corpora, the overall concreteness score showed an upward trend between 1850 and 2000, with the linear regression estimate of the time (by year) slope predicting an increase of 9% in average type concreteness over 150years. Meanwhile, they noted that their database can be used to track changes in the concreteness of individual words (e.g., semantic bleaching of the word *disaster*). While Snefjella et al. (2019) used the SentProp algorithm specifically to evaluate diachronic changes of word concreteness in historical sub-corpora, in our view the algorithm can be extended to examine L2 learners’ development over time or differences across different proficiency levels in terms of not only word concreteness but also other LPP dimensions. Specifically, this algorithm allows us to assess changes in the degree of different LPP dimensions with which L2 learners use the same words as their proficiency levels improve, much in the same fashion it allowed Snefjella et al. (2019) to evaluate diachronic changes in word concreteness. The current study explores the possibility of extending the algorithm to the assessment of five LPP dimensions and to the evaluation of differences among sub-corpora representing L2 learners at different proficiency levels.

## Methodology

### Research Questions

The present study proposes a new semantic similarity algorithm based on that of Snefjella et al. (2019) for estimating the degree of different LPP dimensions in L2 production and uses the algorithm to address the following three research questions:

1. What level of validity can the algorithm achieve for estimating the degree of different LPP dimensions in L2 production?
2. What between-proficiency relationships and cross-proficiency differences exist in the degree of different LPP dimensions in L2 production?
3. What relationships are there among different LPP dimensions in L2 production?

### Corpus

The corpus used in the current study was the EFCAMDAT2 (Huang et al., 2018), one of the largest publicly available learner corpora. EFCAMDAT2 contains 1million assignments (totaling 83 million words) submitted by 174,000 English learners from around the world for coursework on *Englishtown*,^1^ Education First’s online school. Learners were placed into one of 16 proficiency levels initially through a placement test and subsequently through successful progression in coursework. Coursework at each level consisted of eight units offering various types of receptive and productive tasks, and learners submitted a written assignment in response to a writing prompt at the end of each unit, such as writing an email, a summary of a text, or an argumentative essay. The 16 levels correspond to the six levels in the Common European Framework of Reference for Languages (CEFR) as follows: A1 “beginner” (levels 1–3), A2 “elementary” (levels 4–6), B1 “intermediate (levels 7–9), B2 “upper intermediate” (levels 10–12), C1 “advanced” (levels 13–15), and C2 “proficiency” (level 16). Following Römer (2019), we included all texts produced by learners from the levels A1 through C1 in the current study but excluded those by learners at the C2 level, due to the relatively small number of texts in that category. Table 1 summarizes the composition of the five EFCAMDAT2 sub-corpora used in the current study.

**Table 1:** Overview of the five Education First-Cambridge Open Language Database (EFCAMDAT2) sub-corpora used in this study.

Proficiency level	Texts	Learners	Tokens	Word embeddings
A1	625,985	103,742	28.8M	40,471
A2	307,996	52,734	24M	40,464
B1	168,361	32,852	18.4M	27,651
B2	61,329	13,951	9.3M	18,684
C1	14,698	2,839	2.8M	11,592

### LPP Dimension Score Computation

The current study adapted and extended the algorithm and procedure proposed by Snefjella et al. (2019) for measuring diachronic change in word concreteness to compute LPP dimension scores for the five EFCAMDAT2 sub-corpora representing L2 learners of different proficiency levels. This section describes how our algorithm was implemented.

#### Word Embeddings

Word embeddings are vectors or numeric representations of words that capture their meanings or contexts of use, constructed in such a way that semantically similar or related words have vectors that are spatially proximate in a multi-dimensional semantic space. They have been widely used in natural language processing, cognitive science, and language research (Bakarov, 2018) and have been shown to be effective for inferring the psycholinguistic properties of words (Paetzold and Specia, 2016). A highly predictive algorithm for learning word embeddings from raw corpora is word2vec (Mikolov et al., 2013). More recent methods such as BERT and ELMo have also been shown to be able to train contextual word embeddings with robust ambiguity resolution capabilities (Devlin et al., 2018; Peters et al., 2018).

Following Snefjella et al. (2019), we used *FastText* (Bojanowski et al., 2016), an algorithm derived from word2vec that considers morphological information and that is suitable for small corpora, to transform each sub-corpus into a database of word embeddings with 300 dimensions. The database of word embeddings for each sub-corpus included vectors for all words occurring in the sub-corpus, with each vector representing the co-occurrences of a word with other words.

#### The Sentiment Propagation Algorithm

Snefjella et al. (2019) used the SentProp algorithm from the SocialSent package (Hamilton et al., 2016a) to compute concreteness scores for each word in a sub-corpus. The scores for individual words were then used to compute a concreteness score for the entire sub-corpus.

The steps that the algorithm took can be summarized as follows. First, as mentioned above, the *FastText* algorithm was used to derive word embeddings with 300 dimensions from the sub-corpus. Second, a weighted lexical graph was constructed, in which the nodes were words in the sub-corpus, and each node was connected to its *n* nearest semantic neighbors based on the strength of semantic similarity^2^. The strength of semantic similarity between two words was calculated as the *cosine distance* between the two vectors representing those words, which would be a number between 0 and 1, with a higher number corresponding to greater semantic similarity. The semantic similarity strength was also used to weight the edges connecting two nodes. Third, after the graph was constructed, semantic similarities were transformed into probabilities, and a transition matrix describing the probabilities of randomly moving from one word to its neighbors was computed (Hamilton et al., 2016b). Fourth, the SentProp algorithm performed random walks on the transition matrix, using a set of concrete and abstract seed words (see discussion below) as starting points. These walks generated two probability distributions: one representing the proportion of walks landing on each word from the concrete seed words and the other representing the proportion of walks landing on each word from the abstract seed words. To eliminate any bias introduced by a specific seed word, SentProp repeated this procedure using randomly sampled subsets of the seed words. For each random subset of seed words and for each word in the sub-corpus, the values in the two probability distributions were recorded—*a* from abstract seed words and *c* from concrete words—and a concreteness score for the word is computed as *c/*(*c+a*), which was a value between 0 and 1, with a higher score corresponding to a higher degree of concreteness. Finally, the concreteness score for a word was computed as the average of the concreteness scores obtained with all random subsets of seed words. The final concreteness score of a sub-corpus was then the mean of the final concreteness scores of the words in the sub-corpus.

Consider a dramatically simplified example in which we set *n* to 2, and the two nearest semantic neighbors of “table” are “chair” and “furniture,” in that order, as determined by the *cosine* distance between the vector representing “table” and those representing other words. In the weighted lexical graph, “table” will be connected to “chair” and “furniture” with an edge weighted by the semantic similarity between them. In the transition matrix, the probabilities of moving from table to chair and furniture will add up to 1, with a higher probability to move to “chair” because of its stronger semantic strength with “table.” We will leave out the details on semantic similarity weighting and transition matrix computation here (see Hamilton et al., 2016b for details) as they do not affect the conceptual understanding of the algorithm. Now, let us assume that two concrete (e.g., “tree” and “cat”) and two abstract (e.g., “responsibility” and “principle”) seed words are used. We will not do any random sampling of the seed words here given the small number of seed words used but will perform 100 random walks on the transition matrix from each of the four seed words. One random walk starting from “tree” will look as follows: The first step will take us from “tree” to one of its two nearest neighbors (say “wood”), the second step from “wood” to one of its two nearest neighbors (say “furniture”), and so on and so forth, until a specified number of steps (say 2000) have been taken. The words traversed in the walk are recorded, and the next random walk is then performed. Among the 100 random walks, the probability of moving from any word on the path to one of its two neighbors will be determined by the transition probabilities in the transition matrix. In general, a random walk from a concrete seed word will likely land on more concrete words than abstract words, given that concrete words are more likely to have other concrete walks as their nearest neighbors. The opposite is true for a random walk from an abstract seed word. When 100 walks have been performed from all four seed words, the proportion of walks from the two concrete seed words and the two abstract words landing on “table” can be calculated as *c* (say 0.64) and *a* (say 0.16), respectively. The concreteness score of “table” can then be calculated as *c/*(*c+a*)=0.64/(0.64+0.16)=0.8. The concreteness score of the corpus will be the mean of the concreteness scores of all words in the corpus.

This algorithm has three potential advantages over the algorithm adopted in Coh-Metrix and TAALES: It takes into account the frequency of occurrence of the words in each sub-corpus, its coverage is not limited to content words or words covered by databases of rating norms, and it produces a concreteness score for a sub-corpus that reflects how words are used in the sub-corpus (Snefjella et al., 2019). This last point is especially attractive and relevant to us. Although different learner groups may use the same word, their perceptions of the degree of concreteness of the word may vary. This variation can be dynamically captured by the semantic similarity algorithm.

The current study extended the semantic similarity algorithm to estimate scores of five LPP dimensions^3^ of each of the five EFCAMDAT2 sub-corpora being analyzed. While the SentProp algorithm could estimate the degree of a specific LPP dimension of a sub-corpus by analyzing all word embeddings derived from the sub-corpus, Snefjella et al. (2019) chose two sets of target words for concreteness estimation: a set of target words that occurred five or more times in each decade, and a set of words that occurred 50 or more times in each decade, to facilitate cross-decade comparison. In the current study, for each comparison made, we computed the LPP dimension scores for each sub-corpus using all words shared by all sub-corpora being compared. In what follows, we discuss how seed words were selected for the five LPP dimensions.

#### Seed Words

Snefjella et al. (2019) selected 15 concrete and 15 abstract words as seed words. The seed words all occurred frequently (over 500 times) in each decade between 1850 and 2000 and were all rated either extremely concrete (>4.9) or extremely abstract (<2.2) on a five-point scale in Brysbaert et al.’s (2014) database.

The current study also used the criteria of frequency and extreme ratings for seed word selection. Additionally, we required the seed words to be content words that reflect the overall content of the corpora as well. Specifically, we selected 40 seed words for each of the five LPP dimensions as follows. First, we identified a list of words that were shared among the word embeddings derived from the five EFCAMDAT2 sub-corpora and that occurred at least 10 times in each sub-corpus. Second, we used topic modeling (Blei et al., 2003) to obtain a list of the top 1,000 words that best represent the “topics” or overall content of EFCAMDAT2 as a whole. This was done to mitigate the potential effects of tasks on the vocabulary used in the corpus, as previous studies have reported a significant effect of tasks on measures of linguistic complexity in the EFCAMDAT (e.g., Alexopoulou et al., 2017). Grün and Hornik’s (2011) R package, topicmodels, which estimates topic words using Latent Dirichlet allocation (LDA) and Gibbs sampling, was used for this purpose. Third, the words that were on both lists generated from the first two steps became our candidate seed words. Finally, for each of the five LPP dimensions considered, we identified the 20 highest and 20 lowest rated candidate seed words in the largest available database of rating norms for that dimension. For concreteness, Brysbaert et al.’s (2014) database was used. For the other four dimensions, the Glasgow norms were used. This four-step procedure ensured that the seed words identified (see Supplementary Material) were frequently used in all five EFCAMDAT2 sub-corpora, were representative of the overall content of EFCAMDAT2, and had a high degree of reliability in their ratings. Table 2 illustrates the LPP dimension scores computed for the word *egg* by the algorithm with these seed words in the five EFCAMDAT2 sub-corpora. We do not intend to delve into an in-depth qualitative analysis of how the different uses of the word across different proficiency levels may have given rise to the fluctuations in its LPP dimension scores at this point, but we note in passing that lower-proficiency learners have used the word primarily in its basic sense (e.g., *I do not like eggs*), which is acquired early, familiar, concrete, imageable, and small in semantic size, while advanced learners have acquired additional meanings of the word, which may be less familiar, more abstract, less imageable, and possibly semantically larger (e.g., *I try to build a nest egg*, *bold architecture conveying balconies as eggs*, etc.).

**Table 2:** Sample LPP dimension scores computed by the algorithm for the word *egg* in the five EFCAMDAT2 sub-corpora.

Dimension	A1	A2	B1	B2	C1	Mean	Human Rating
FAM	0.95	0.90	0.82	0.73	0.48	0.78	6.3/7 = 0.90
IMAG	0.96	0.92	0.86	0.86	0.67	0.85	6.65/7 = 0.95
SIZE	0.03	0.12	0.12	0.17	0.18	0.13	2.03/7 = 0.29
AoA	0.20	0.29	0.33	0.22	0.53	0.31	1.89/7 = 0.27
CONC	0.82	0.91	0.90	0.84	0.67	0.83	4.97/5 = 0.99

FAM, familiarity; IMAG, imageability; SIZE, semantic size; AoA, age of acquisition; CONC, concreteness. The denominator in the human rating indicates the maximum of the rating scale.

### Statistical Analysis

After computing the LPP dimension scores for the five sub-corpora, we used a combination of statistical methods to address the three research questions. To address the first research question, we computed the correlations between algorithm-generated LPP dimension scores and human ratings in the databases used in the current study.

To address the second research question, we employed Bayesian regression analysis (Gelman et al., 2013; Bürkner, 2017; McElreath, 2018) to determine the extent to which the LPP dimension scores of one or more lower levels (predictors) could predict the scores of a higher level (response variable)^4^ and the Euclidean and Manhattan distances to assess differences among the LPP dimensions scores of different sub-corpora. Snefjella et al. (2019) used the correlations between concreteness scores for adjacent decades as a measure of validity of the algorithm-generated scores, with the assumption that adjacent decades should not see dramatic changes in word concreteness. Given that we cannot presume the same about adjacent proficiency levels, we did not use a similar correlation analysis as a validity measure. Rather, we intended to empirically test the extent to which cross-proficiency differences or changes in LPP dimension scores may be gradual or abrupt. The Bayesian regression analysis has been shown to be superior to correlation analysis when it comes to revealing whether predictors have significant effects on response variables; it has also been shown to be potentially more effective than other regression models (e.g., generalized mixed regression models) on small-scale data (Norouzian et al., 2018).

To address the third research question, we first used correlation analysis to obtain a preliminary sense of the relationships among the scores of different LPP dimensions. We then employed hierarchical clustering (Kaufman and Rousseeuw, 2009) to visualize and further scrutinize the relationships among those dimensions and to compare how those relationships differ among L1 and L2 speakers.

## Results

### Validity of Algorithm-Computed LPP Dimension Scores

To obtain a sense of the validity of the LPP dimension scores computed by the algorithm in relation to the respective psychological constructs, we calculated the Spearman’s correlations between algorithm-computed LPP dimension scores and human ratings in the databases used in the current study, namely Brysbaert et al. (2014), and Scott et al. (2019). Specifically, for each LPP dimension, we first calculated the mean of the dimension scores for each target word across the five EFCAMDAT2 sub-corpora. We then calculated the correlations between the aggregated LPP dimension scores of the target words and their human ratings in the corresponding databases.

**Figure 1:**
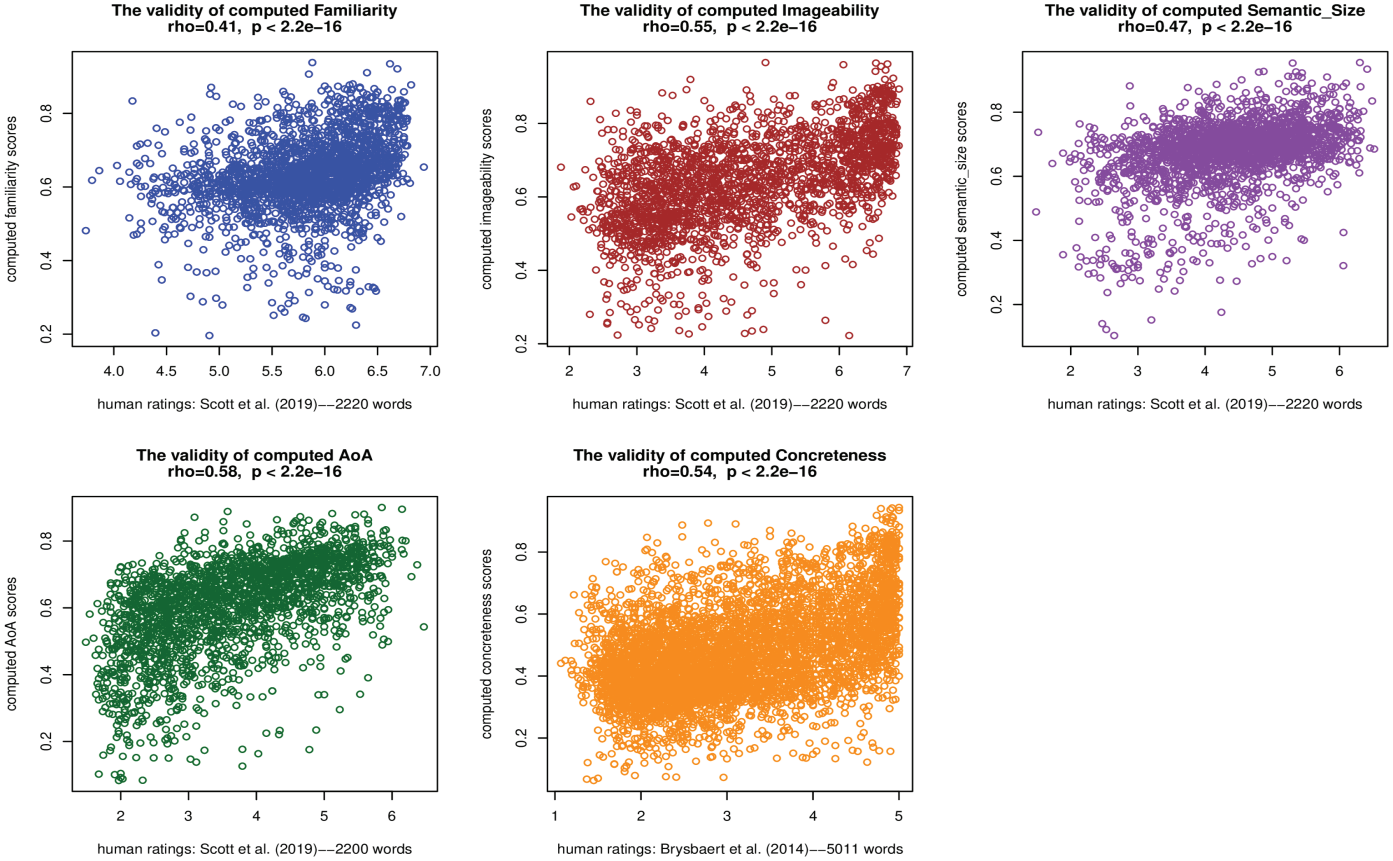
Spearman’s correlations between aggregated computed LPP dimension scores and human ratings in the EFCAMDAT2.

Figure 1 displays the Spearman’s correlations between the aggregated computed LPP dimension scores and human ratings in the EFCAMDAT2. The correlation coefficients ranged from 0.41 for familiarity to 0.58 for AoA, and all correlations were statistically significant (*p*<2.2e-16). Overall, the significant medium-to-large correlations (Cohen, 1992) suggested a good level of validity of the LPP dimension scores computed by the algorithm. Additionally, Supplementary Material B summarizes the Spearman’s correlations between computed LPP dimension scores and human ratings in the five EFCAMDAT2 sub-corpora. For each LPP dimension, the correlations for the different proficiency levels were close to the overall correlation. We return to a discussion of these results in Discussion section.

### Relationships and Differences Among L2 Learners at Different Proficiency Levels

This section reports results regarding the relationships and differences among L2 learners at different proficiency levels in terms of the LPP dimension scores of their written production. As discussed earlier, the EFCAMDAT2 was divided into five sub-corpora representing L2 English learners at five proficiency levels (A1, A2, B1, B2, and C1), respectively.

**Figure 2:**
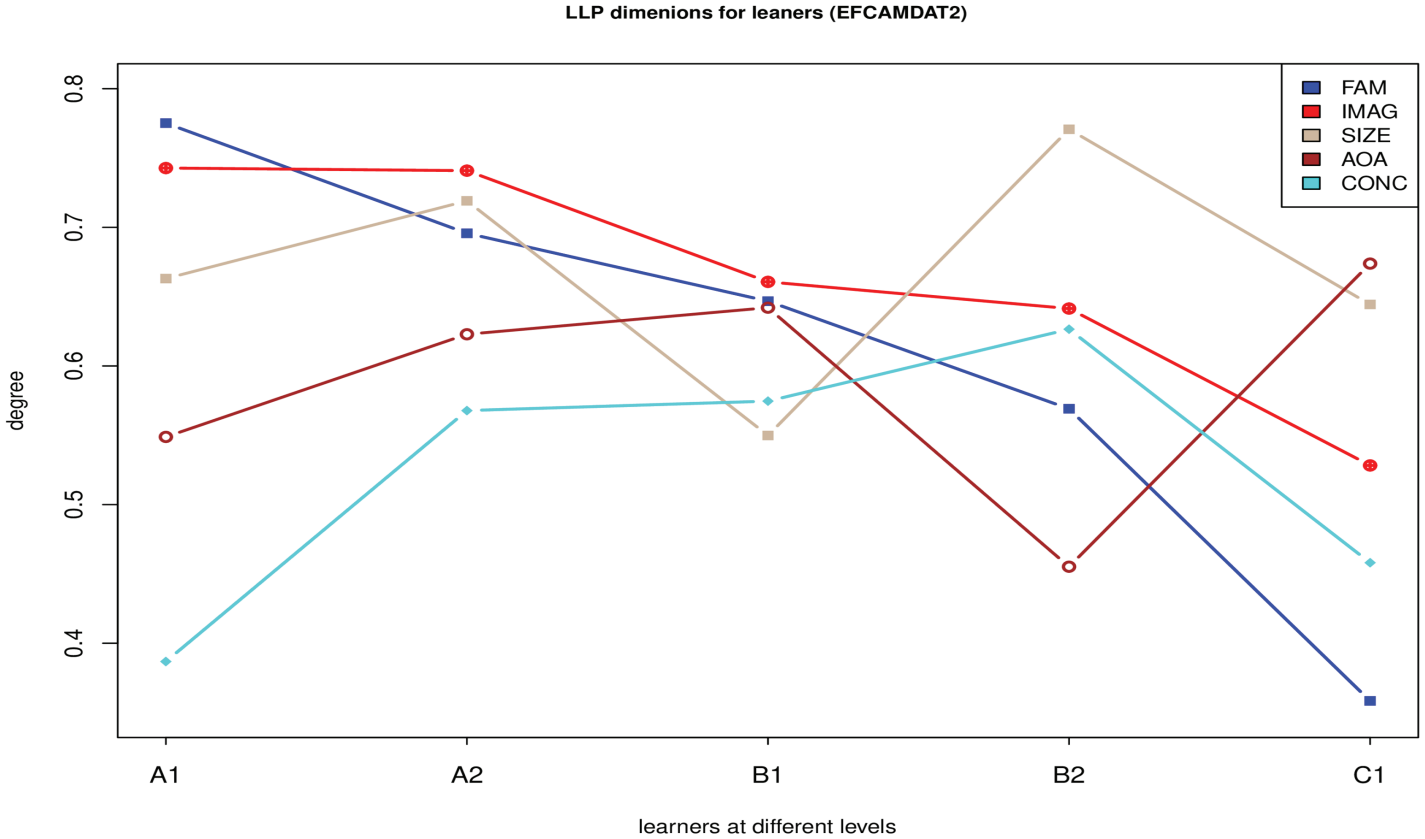
LPP dimension scores of the five sub-corpora of the EFCAMDAT2. FAM, familiarity; IMAG, imageability; SIZE, semantic size; AoA, age of acquisition; CONC, concreteness.

Figure 2 displays the LPP dimension scores of the five sub-corpora. Familiarity and imageability decreased linearly from A1 to C1, while the other three LPP dimensions did not increase or decrease linearly across the five proficiency levels, with each level having two LPP dimension scores that were either the highest or the lowest among all five sub-corpora.

Table 3 summarizes the Bayesian regression results on the five EFCAMDAT2 sub-corpora. In Models 1 to 4, the scores of a lower level were used to predict the scores of the next level. In all four models, the fixed effect estimates (FEEs) for the lower-level scores were consistently positive and significant. In Model 5, the scores of the two lowest levels (i.e., A1 and A2) were used to predict the scores of the B1 level. In this model, the FEEs for A2 scores were consistently positive and significant. However, the FEEs for A1 scores were significant but negative for all five LPP dimensions (marked in blue in Table 3). In Model 6, the scores of the three lowest levels (i.e., A1, A2, and B1) were used to predict the scores of the B2 level. In this model, the FEEs for B1 scores were significant and positive for all LPP dimensions except semantic size. The FEEs for A1 scores were significant and negative for all LPP dimensions. The FEEs for A2 scores were significant and positive for four LPP dimensions and non-significant for imageability. In Model 7, the four lower level scores were used to predict the scores of the highest level (i.e., C1). In this model, the FEEs for B2 scores were significant and positive for all LPP dimensions. However, the FEEs for B1, A2, and A1 scores, while significant for all LPP dimensions, were positive for some dimensions and negative for others. Overall, these results indicated that the scores of a higher level were best predicted positively by those of the adjacent lower level.

**Table 3:** Bayesian regression results on the five EFCAMDAT2 sub-corpora (*N*=7883).

Model	FAM	IMAG	SIZE	AoA	CONC
#1: A1→A2	0.95 (0.94, 0.95)	0.77 (0.77, 0.78)	0.71 (0.71, 0.713)	0.76 (0.75, 0.76)	0.92 (0.91, 0.923)
#2: A2→B1	0.94 (0.94, 0.94)	0.77 (0.77, 0.77)	0.99 (0.98, 0.99)	0.94 (0.93, 0.94)	0.8 (0.795, 0.8)
#3: B1→B2	0.8 (0.8, 0.81)	0.99 (0.99, 0.997)	0.72 (0.71, 0.723)	0.76 (0.762, 0.769)	0.66 (0.654, 0.66)
#4: B2→C1	0.74 (0.74, 0.75)	0.46 (0.46, 0.463)	0.55 (0.55, 0.553)	0.51 (0.51, 0.515)	0.79 (0.785, 0.79)
#5: A1+A2→B1	A2: 1.61 (1.59, 1.64)	A2: 0.8 (0.78, 0.8)	A2: 1.63 (1.6, 1.67)	A2: 1.25 (1.24, 1.26)	A2: 0.8 (0.78, 0.8)
	A1: −0.64 (−0.66, −0.62)	A1: −0.02 (−0.028, −0.011)	A1: −0.27 (−0.31, −0.237)	A1: −0.24 (−0.24, −0.23)	A1: −0.02 (−0.028,-0.011)
#6: A1+A2+B1→B2	B1: 0.3 (0.28, 0.32)	B1: 1.03 (1.02, 1.04)	B1: −0.206 (−0.210,-0.202)	B1: 0.49 (0.45, 0.52)	B1: 1.45 (1.44, 1.46)
	A2: 1.56 (1.52, 1.6)	*A2: 0.005 (−0.002,0.012)	A2: 1.3 (1.3, 1.31)	A2: 1.03 (0.99, 1.08)	A2: −0.6 (−0.61, −0.59)
	A1: −1.04 (−1.06, −1.01)	A1: −0.025 (−0.027, −0.021)	A1: −0.22 (−0.22, −0.21)	A1: −0.6 (−0.61, −0.59)	A1: −0.04 (−0.046, −0.039)
#7: A1+A2+B1+B2→C1	B2: 1.11 (1.09, 1.13)	B2: 0.08 (0.04, 0.13)	B2: 1.32 (1.29, 1.35)	B2: 0.19 (0.17, 0.21)	B2: 1.11 (1.09, 1.13)
	B1: −0.55 (−0.57, −0.53)	B1: 1.2 (1.15, 1.24)	B1: 0.26 (0.25, 0.27)	B1: 1.36 (1.32, 1.39)	B1: −0.52 (−0.56, −0.48)
	A2: 0.9 (0.85, 0.95)	A2: −0.58 (−0.6, −0.57)	A2: −0.57 (−0.62, −0.53)	A2: −1.41 (−1.46, −1.36)	A2: 0.1 (0.09, 0.12)
	A1: −0.65 (−0.67, −0.61)	A1: −0.047 (−0.052,−0.04)	A1: −0.21 (−0.23, −0.2)	A1: 0.29 (0.28, 0.31)	A1: 0.16 (0.15, 0.17)

In each model, the response variable appears to the right of “→”, and the predictor(s) appear to the left of “→”. “+” indicates multiple predictors in the model. The result cells provide the fixed effect estimates (FEE) and their corresponding 95% Posterior Credible Intervals (PCI) for the predictors. An FEE is significant if the 95% PCI does not contain 0. Non-significant FEEs are marked with asterisk.

Table 4 summarizes the Euclidean and Manhattan distances between the LPP dimension scores of each pair of sub-corpora of the EFCAMDAT2. Results from both distance measures consistently indicated that, among all pairs of adjacent levels, the distance between A1 and C1 scores was the greatest, while that between A2 and B1 scores was the smallest. Overall, these results are consistent with those presented in Figure 2.

**Table 4:** Euclidean and Manhattan distances among the five EFCAMDAT2 sub-corpora.

	Euclidean distance					Manhattan distance				
	A1	A2	B1	B2	C1	A1	A2	B1	B2	C1
A2	0.22	0				0.39	0			
B1	0.28	0.19	0			0.6	0.32	0		
B2	0.36	0.25	0.3	0		0.75	0.5	0.56	0	
C1	0.5	0.42	0.35	0.38	0	0.85	0.79	0.66	0.84	0

### Relationships Among Different LPP Dimensions

**Figure 3:**
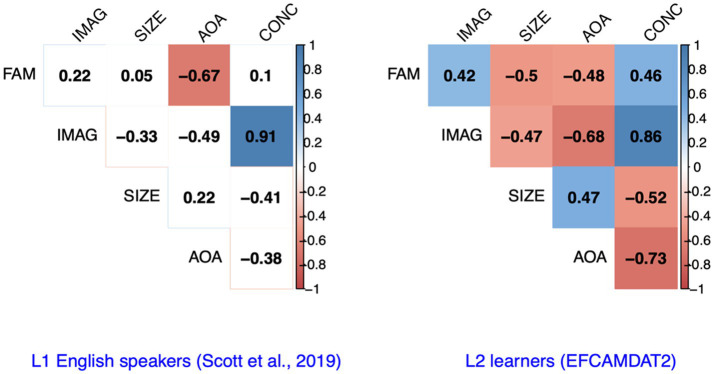
Correlations among the scores of different LPP dimensions for L1 English speakers and L2 English learners. FAM, familiarity; IMAG, imageability; SIZE, semantic size; AoA, age of acquisition; CONC, concreteness.

To assess the relationships among the five LPP dimensions manifested in our data, we computed the correlation coefficients among the scores of different LPP dimensions for the target words considered in the EFCAMDAT2. For comparison purposes, we drew on results reported on the correlations among different LPP dimensions for L1 English speakers by Scott et al. (2019). As shown in Figure 3, for L1 speakers, imageability and concreteness were significantly positively correlated with each other, while AoA and familiarity were significantly negatively correlated with each other (*p*<0.05). For L2 learners, however, all five LPP dimensions were significantly correlated with each either positively or negatively. The directions of the correlations among the five LPP dimensions were largely consistent between L1 speakers and L2 learners, with the exception of the correlation between familiarity and semantic size, which was positive (but trivial) for L1 speakers and negative for L2 learners.

**Figure 4:**
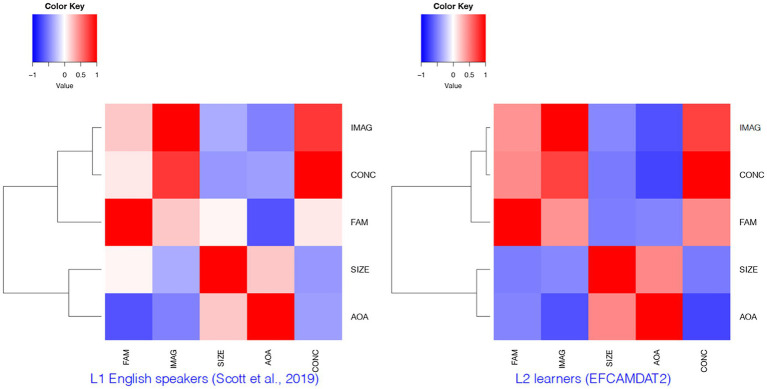
Clusters of LPP dimensions for L1 English speakers and L2 learners. FAM, familiarity; IMAG, imageability; SIZE, semantic size; AoA, age of acquisition; CONC, concreteness.

To further examine the relationships among the LPP dimensions and how these relationships differ among L1 and L2 speakers, we performed hierarchical clustering on the EFCAMDAT2 using the *heatmap* function in R, which produced both a dendrogram and a heatmap. The heatmap was a color-coded table in which the darkness of the colors corresponds to the strength of correlation between different LPP dimensions, with blue and red indicating positive and negative correlations, respectively. For comparison purposes, we again drew on results reported for L1 English speakers by Scott et al. (2019). These results of the cluster analysis are presented in Figure 4. A quick scanning of the dendrograms revealed fairly consistent clusters of the LPP dimensions for L1 speakers and L2 learners. The heatmaps allow us to visualize the differences in the strength of the correlations among the LPP dimensions between L1 speakers and L2 learners, such as the darker red color for the correlation between AoA and imageability for L2 learners than for L1 speakers.

## Discussion

### Validity of Algorithm-Generated LPP Dimension Scores

Overall, the LPP dimension scores generated by the semantic similarity algorithm for the learner corpus demonstrated significant medium-to-large correlations with human ratings, indicating good validity of the algorithm-generated LPP dimension scores in relation to the corresponding psychological constructs. Two issues are worth noting.

First, the correlations observed in the current study were lower than the correlation (*ρ*=0.70, *p*<0.001) reported by Snefjella et al. (2019) between the aggregated algorithm-computed concreteness scores for their diachronic English corpus and human ratings in Brysbaert et al. (2014). For a more direct comparison, the correlation between the aggregated algorithm-computed concreteness scores for the EFCAMDAT2 and human ratings in Brysbaert et al. (2014) was 0.48 (*p*<2.2e-16). The difference in corpus size, which has been shown to impact the performance of word embedding models (e.g., Altszyler et al., 2016; Crossley et al., 2017), may have contributed to this difference. The corpus used in Snefjella et al. (2019) contained 400 million words, while the EFCAMDAT2 contained 83 million words. In addition, Snefjella et al. (2019) correlated algorithm-computed scores of a corpus of L1 English texts to human ratings produced by L1 speakers, whereas the current study correlated algorithm-computed scores of the corpus of L2 English texts to those ratings. The lower correlations may also to some extent reflect differences between L1 and L2 speakers’ perceptions of the LPP dimensions of English words and their lexical usage.

Second, while the correlations observed for all five LPP dimensions were significant, the effect sizes varied somewhat. Specifically, AoA and imageability achieved large correlation coefficients, while the other three dimensions (i.e., familiarity, semantic size, and concreteness) achieved medium correlation coefficients. Further research is needed to confirm whether such variation should be interpreted as the algorithms’ differential ability to capture different LPP dimensions or as varied degrees of differences of L2 learners’ perceptions of different LPP dimensions from L1 speakers’ perceptions and ratings of those dimensions.

### Differences Among Learners at Different Proficiency Levels

With respect to between-proficiency relationships, results from the Bayesian regression analyses indicated that the LPP dimension scores of lower proficiency levels were generally predictive of scores of higher proficiency levels. Significant positive effects were consistently found in all models in which the scores of a lower level were used to predict the scores of the adjacent higher level in both corpora. Snefjella et al. (2019) used strong correlations between the concreteness scores for adjacent decades as a measure of validity of algorithm-computed scores, based on the assumption that diachronic changes in word concreteness should be a gradual process. We interpret these results as confirming that the LPP dimension scores of adjacent proficiency levels do not deviate from each other dramatically but are strongly related to each other.

In models where the scores of two or more lower levels were used to predict the scores of a higher level, there was some variation in the effects observed for the lower-level scores across the LPP dimensions. The scores of the adjacent lower level consistently positively predicted the scores of the higher level across all dimensions and all models (with the exception of semantic size in Model 6), while the non-adjacent lower levels showed a mixture of positive, negative, or non-significant effects for different LPP dimension in different models. The results offer additional evidence for stronger relationships between scores of adjacent levels than among non-adjacent levels.

With respect to cross-proficiency differences, results from the distance analysis consistently indicated that scores of the adjacent levels crossing the A, B, and C categories were consistently more distant from each other than adjacent levels within a category. The greatest distance was found between A1 and C1, followed by A2 and C1. These results indicate that while L2 learners at different proficiency levels used the target words in different ways in terms of different LPP dimensions, the differences were more marked across CEFR proficiency bands than within CEFR bands. The results also suggest that the differences were even larger between intermediate and advanced levels than between beginner and intermediate levels.

As mentioned earlier, a major methodological difference between the current study and previous L2 studies of LPP dimensions is the dynamic vs. static approach to assessing the degree of different LPP dimension of different sub-corpora. The static approach assumes that between-proficiency differences in the degree of an LPP dimension can be captured by comparing the average ratings of the words that appear in sub-corpora representing different proficiency levels and that are covered by a database for that LPP dimension. For example, Salsbury et al. (2011) and Crossley and Skalicky (2019) reported that longitudinally, L2 lexical production became less concrete and more abstract over time. This did not mean that the degree of concreteness of the same words used by L2 learners decreased as a function of time, but that L2 learners used a higher proportion of words with higher concreteness ratings in the psycholinguistic database later in the study period. Using a dynamic approach to assessing diachronic changes in word concreteness, Snefjella et al. (2019) showed that the degree of concreteness of the same words may change over time. In a similar spirit, our dynamic analysis showed that learners across different proficiency levels used the same target words with different degrees of concreteness as well as other LPP dimensions.

The concreteness scores of the target words increased from A1 through B2 but decreased at the C1 level. Largely consistent with the findings reported in the previous research (Salsbury et al., 2011; Crossley and Skalicky, 2019; De Wilde et al., 2020), these findings indicate a general tendency for learners to use words in more abstract ways as their proficiency levels increase, but the decrease in concreteness is not linear at the higher proficiency level. Salsbury et al. (2011) and Crossley and Skalicky (2019) reported that word imageability also decreased as a function of time for L2 learners. Our results largely confirmed this finding. In the EFCAMDAT2, imageability was higher for the A1, A2, and B1 levels and lower for the B2 and C1 levels. Salsbury et al. (2011) and Crossley and Skalicky (2019) found no evidence that learners used fewer familiar words as they developed. Different from these findings, our results indicated a linear decrease in familiarity from A1 to C1. These results thus suggest a trend toward greater use of the same words in less familiar ways or contexts as learners become more proficient. AoA increased from A1 through B1 but then dropped to the lowest point at B2 before increasing again at C1. This nonlinear pattern is not inconsistent with the results reported by De Wilde et al. (2020), who found a positive effect of AoA on proficiency for learners in the low-proficiency group but a negative effect on proficiency for learners in the high-proficiency group. De Wilde et al. (2020, p. 372) considered the higher scores for words learned later in L1 English among lower proficiency learners surprising and argued that “mechanisms or phenomena that determine the AoA of L1 words may not have much direct relation to those that determine the very initial stages of L2 rod learning.” Semantic size exhibited no clear increasing or decreasing trend across proficiency levels, with the B2 level demonstrating the highest score and the B1 level demonstrating the lowest score. This result suggests that semantic size does not have a linear association with L2 proficiency and that other factors (e.g., tasks) may have contributed more to the variation in the scores of this LPP dimension.

Given that we computed the LPP dimension scores for the five sub-corpora using the same set of target words, the substantial cross-proficiency differences in the LPP dimension scores in L2 production observed suggest that L2 learners at different proficiency levels may perceive the LPPs of the same words differently and accordingly use them in rather different ways. The substantial consistency between our findings and those reported in previous studies also offers additional support for the validity of our algorithm for assessing the LPPs in L2 production.

### Relationships Among Different LPP Dimensions

Results of the correlation analysis and cluster analysis revealed the following similarities in the relationships among different LPP dimensions between L1 English speakers and L2 learners. First, concreteness and imageability were significantly positively correlated and formed a first-level cluster. The close relationship between these two dimensions observed offers support for the imageability hypothesis, which predicts that language users are more likely to generate images or imaginations for concrete words than for abstract words (Paivio et al., 1968; Richardson, 1975; Kousta et al., 2011). Second, semantic size and AoA formed another first-level cluster, with a positive correlation with each other and negative correlations with both imageability and concreteness. Third, familiarity formed a second-level cluster with concreteness and imageability, with positive correlations with both of them and negative correlations with AoA and semantic size (with the exception of a trivial positive correlation with semantic size for L1 speakers). The mostly consistent results regarding the directions of the correlations among the five LPP dimensions and the clusters they formed suggest that L1 English speakers and L2 learners perceived these relationships among the psychological constructs in substantially similar ways.

Meanwhile, the correlation analysis also revealed substantial differences in the degree of significance and strength of the correlations among the LPP dimensions between L1 speakers and L2 learners. Only two pairs of LPP dimensions were significantly correlated for L1 speakers, indicating a high degree of independence of the psychological constructs underlying the LPP dimensions. However, all 10 pairs were significantly correlated for L2 learners, suggesting that L2 learners perceived them more similarly than L1 English speakers, and it is possible that their more limited vocabulary and knowledge of varying word meanings played a role.

## Conclusion

This study extended a semantic similarity algorithm to estimate the degree of five different LPP dimensions in L2 production and used the algorithm to analyze multiple sub-corpora from the EFCAMDAT2 to examine between-proficiency relationships and cross-proficiency differences in the LPPs in L2 production as well as the relationships among different LPP dimensions. The state-of-the-art algorithm allowed us to adopt a dynamic approach to assessing the degree of different LPP dimensions across different EFCAMDAT2 sub-corpora representing different learners at different proficiency levels, bypassing the limitations associated with the static approach adopted in previous L2 studies of LPPs.

Our findings suggested good validity of the LPP dimension scores computed by the dynamic semantic similarity algorithm. The LPP dimension scores of lower proficiency levels were generally predictive of scores of adjacent higher levels, indicating that cross-proficiency changes were overall gradual rather than abrupt. At the same time, overall cross-proficiency differences were larger between intermediate and advanced levels than between beginner and intermediate levels. Familiarity showed a consistent decreasing trend across proficiency levels, and concreteness and imageability showed relatively clear but nonlinear increasing trends across proficiency levels, while AoA and semantic size showed less clear trends of cross-proficiency changes. In terms of the relationships among the LPP dimensions, our findings revealed substantial similarities in the directions of the correlations among the LPP dimensions and the clusters they formed but also substantial differences in the degree of significance and strength of the correlations among the LPP dimensions between L1 speakers and L2 learners.

Overall, this study has provided initial evidence for the validity of the dynamic semantic similarity algorithm for assessing the degree of LPP dimensions in L2 production and contributed to a more comprehensive understanding of the LPPs in written texts produced by L2 learners at different proficiency levels. This method is not intended to replace subjective rating norms commonly used by psycholinguistic researchers but to complement such norms with the ability to bypass coverage issues of such normal, the functionality to evaluate the overall degree of an LPP dimension of a text, and the potential to capture dynamic changes in the degree of an LPP dimension of words. In our future research, we hope to apply the methods proposed in the current study to longitudinal L2 corpora to cross-verify our findings from cross-sectional comparisons. Additionally, it would be highly useful to recruit L2 learners at different proficiency levels to rate a set of English words in terms of different LPP dimensions in ways similar to how LPP scores were obtained from L1 speakers. Such ratings from L2 learners can be used to further evaluate the performance of the algorithm and to shed more light on the differences in the LPPs in L1 and L2 production.

## Author’s Notes

1. Both second and foreign language learners are referred to as L2 learners in the current study.
2. The parameter *n* can be determined based on corpus size, and Snefjella et al. (2019) used 25.
3. While AoA and familiarity may not appear to be as clearly conceptually related to semantic factors as concreteness, imageability, and semantic size, some researchers shown that AoA should be treated as a semantic variable (Van Loon-Vervoorn, 1989), that AoA effects originate from the semantic system (Brysbaert et al., 2000), and that word familiarity and conceptual familiarity/memory are closely related (e.g., Wang and Yonelinas, 2012; Chedid et al., 2019). These findings provide a basis for us to consider AoA and familiarity as semantic factors conceptually and to empirically explore the extent to which the algorithm’s estimation of these two LPP dimensions is valid.
4. The technical details of the Bayesian regression analysis can be found at https://osf.io/jr9hs/.

## Data Availability Statement

The original contributions presented in the study are included in the article/Supplementary Material, and further inquiries can be directed to the corresponding author. The data that support the findings of this study are publicly available from the University of Cambridge at https://philarion.mml.cam.ac.uk/.

## Author Contributions

KS designed this study. KS and XL analyzed the data and wrote this paper. All authors contributed to the article and approved the submitted version.

## Conflict of Interest

The authors declare that the research was conducted in the absence of any commercial or financial relationships that could be construed as a potential conflict of interest.

## Publisher’s Note

All claims expressed in this article are solely those of the authors and do not necessarily represent those of their affiliated organizations, or those of the publisher, the editors and the reviewers. Any product that may be evaluated in this article, or claim that may be made by its manufacturer, is not guaranteed or endorsed by the publisher.

## Footnotes

^1^ www.englishtown.com
