# Supplementary material for: Assessing Lexical Psychological Properties in Second Language Production: A Dynamic Semantic Similarity Approach
Source: Front Psychol. 2021 Sep 23;12:672243. doi: 10.3389/fpsyg.2021.672243 (PMC8495422; doi:10.3389/fpsyg.2021.672243)
Supplement: Supplementary file 1 [file Data_Sheet_1.doc]

**Supplementary Material of “Assessing lexical psychological properties in second language production: A dynamic semantic similarity approach”**

*Kun Sun, Xiaofei Lu*

**A) Seed words**

| **Dimension** | **Seeds words in EFCAMDAT2** |
| --- | --- |
| familiarity | *Positive:* music, fridge, love, hand, phone, food, student, sleep, dog, car, toilet, hair, plate, street, chocolate, smile, body, water, coin, road  *Negative:* mount, panorama, toll, port, mall, merchant, scope, matrix, prophet, symphony, prestige, fleet, heir, disposition, marvel, metropolis, martial, ford, coup, dynasty |
| imageability | *Positive:* bee, hand, apple, bullet, fridge, computer, piano, dog, frog, lion, ambulance, water, car, cow, television, helicopter, pyramid, violin, elephant, chocolate  *Negative:* perception, opinion, tone, fate, mind, least, tendency, use, disposition, ideal, notion, philosophy, personality, affect, abstract, instance, economy, occur, aspect, remind |
| semantic size | *Positive:* universe, war, ocean, university, world, heaven, revolution, planet, empire, life, kingdom, moon, earthquake, disaster, sky, globe, maximum, civilization, giant, mountain  *Negative:* seed, ear, chip, egg, pearl, minimum, mouse, shell, dust, inch, button, pen, zero, bee, rice, moth, worm, penny, ant, grain |
| concreteness | *Positive:* apple, armchair, baby, ball, basket, bat, bed, bike, bird, cage, camera, clock, cup, eagle, ear, elephant, finger, fish, flower, flute  *Negative:* hope, possibility, luck, ideal, fate, responsibility, interpretation, concept, extent, adversity, justice, glory, greed, involvement, theory, accordance, purpose, behalf, privacy, wisdom |
| age of acquisition | *Positive:* prosperity, disposition, legislation, psychologist, metropolis, corporation, adversity, analyst, prestige, velocity, semester, entrepreneur, abundance, cocktail, matrix, competence, forum, premium, dynasty, payroll  *Negative:* finger, mother, sun, sky, colour, eat, bad, cry, bed, apple, name, goodbye, water, grandma, arm, toy, red, boy, yellow, head |

**B) Spearman’s correlations between computed LPP dimension scores and human ratings in the five EFCAMDAT2 sub-corpora**

| **Proficiency level** | **Familiarity** | **Imageability** | **Semantic size** | **Age of Acquisition** | **Concreteness** |
| --- | --- | --- | --- | --- | --- |
| A1 | 0.412 | 0.571 | 0.463 | 0.558 | 0.53 |
| A2 | 0.412 | 0.55 | 0.46.8 | 0.587 | 0.535 |
| B1 | 0.404 | 0.544 | 0.476 | 0.587 | 0.529 |
| B2 | 0.413 | 0.54 | 0.46 | 0.602 | 0.503 |
| C1 | 0.39 | 0.50 | 0.453 | 0.541 | 0.501 |
| Mean | 0.41 | 0.55 | 0.47 | 0.58 | 0.536 |
